# Case Report: Primary dural based diffuse large B-Cell lymphoma in a 14 year-old boy

**DOI:** 10.12688/f1000research.6269.1

**Published:** 2015-03-25

**Authors:** Sunil Munakomi, Binod Bhattarai, Balaji Srinivas, Iype Cherian

**Affiliations:** 1 International Society for Medical Education, College of Medical Sciences, Bharatpur, Chitwan, 44207, Nepal

**Keywords:** Surgical Resection, Primary dural diffuse large B-cell lymphoma, Paediatric cancer, Herniation syndrome, Intracranial lymphoma

## Abstract

Primary dural lymphoma is a subentity of primary leptomeningeal lymphoma which represents 0.1% of all non-Hodgkin’s lymphomas. Only five cases have been reported so far. We report a very rare case of primary dural-based lymphoma in a 14 year-old boy presenting with mass effect. The patient was managed with excision of the lesion and removal of the involved bone. Post-operatively, the patient showed good recovery. He was then referred to the oncology unit for further chemo- and radiation therapy. A high index of suspicion should therefore be kept in order to diagnose the condition in a timely fashion and then plan for appropriate management since diffuse large cell lymphoma has a relatively benign clinical prognosis.

## Introduction

Primary dural diffuse large B-cell lymphoma (DLBCL) is an extremely rare entity with only five cases reported so far ^1^. The symptoms are nonspecific. The main differential diagnosis of the condition remains meningioma ^2^. Currently there is no standard treatment due to a paucity of cases ^3^. A high index of suspicion should be kept in order to diagnose the condition in a timely fashion and then plan for appropriate management since diffuse large cell lymphoma has a relatively benign clinical prognosis ^4^. Here we report a case of a primary dural based DLBCL in a 14 year-old boy presenting with herniation syndrome, who improved after surgical excision and is currently on chemotherapy.

## Case report

A 14 year-old Tharu boy, from Siraha (a remote village in Nepal) presented to our emergency department with a sudden onset altered sensorium which lasted for 1 day. The patient had a history of intermittent headaches and vomiting over the last 3 months. The patient’s parents also noticed significant weight loss and the presence of scalp swelling for the last 2 months. There was no remarkable family history. Previous treatment history revealed that the patient had been taken to India 1 month back, where fine needle aspiration cytology (FNAC) of the scalp lesion in the parietal region had revealed Non-Hodgkin’s lymphoma. The patient party was told the prognosis and advised for chemo- and radiation therapy but this was refused because of their poor financial status and so the family returned back to Nepal.

**Figure 1.**
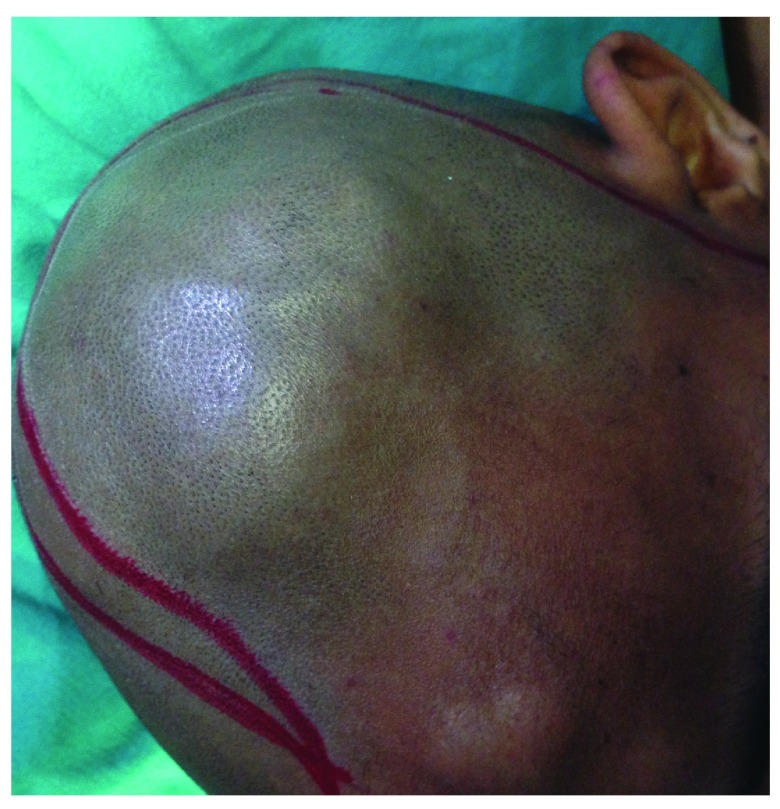
Preoperative image showing frontal and parietal swellings.

**Figure 2.**
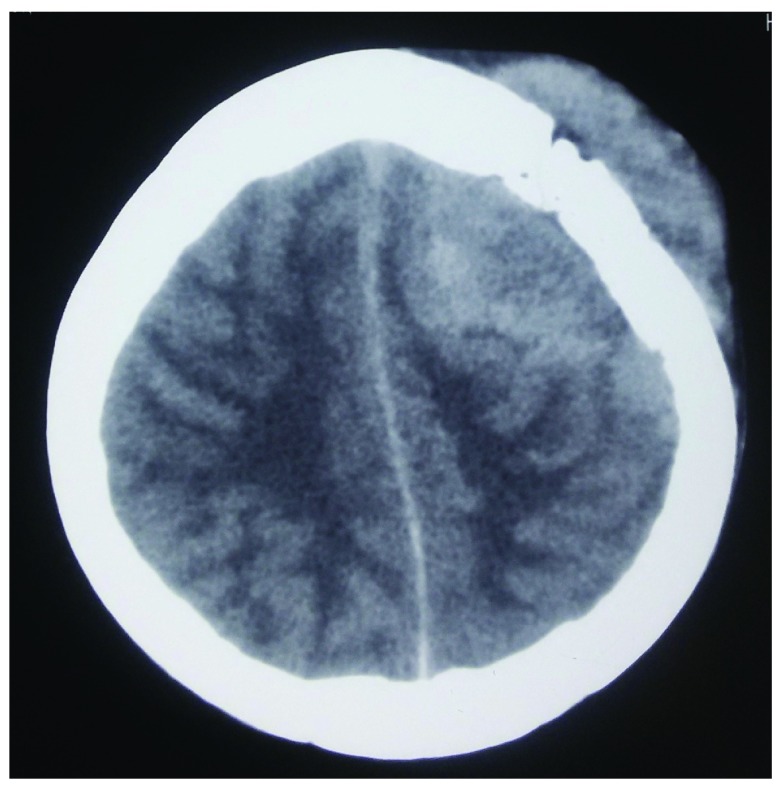
CT image showing hyperintense lesion surrounding the skull bone.

**Figure 3.**
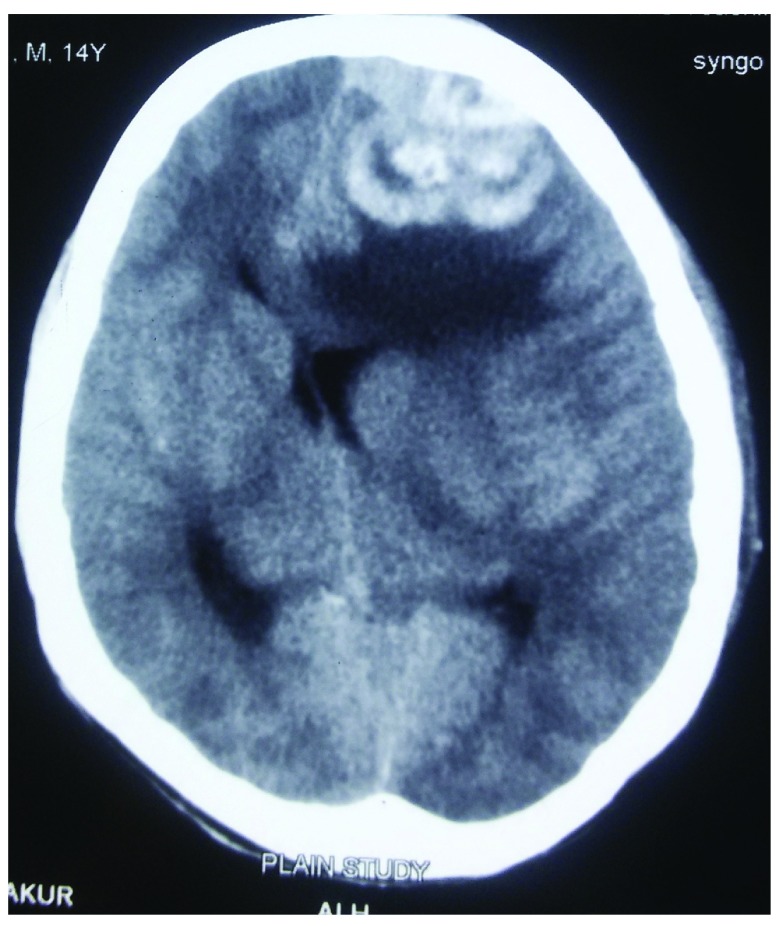
CT image showing the herniation syndrome with gross mass effect.

**Figure 4.**
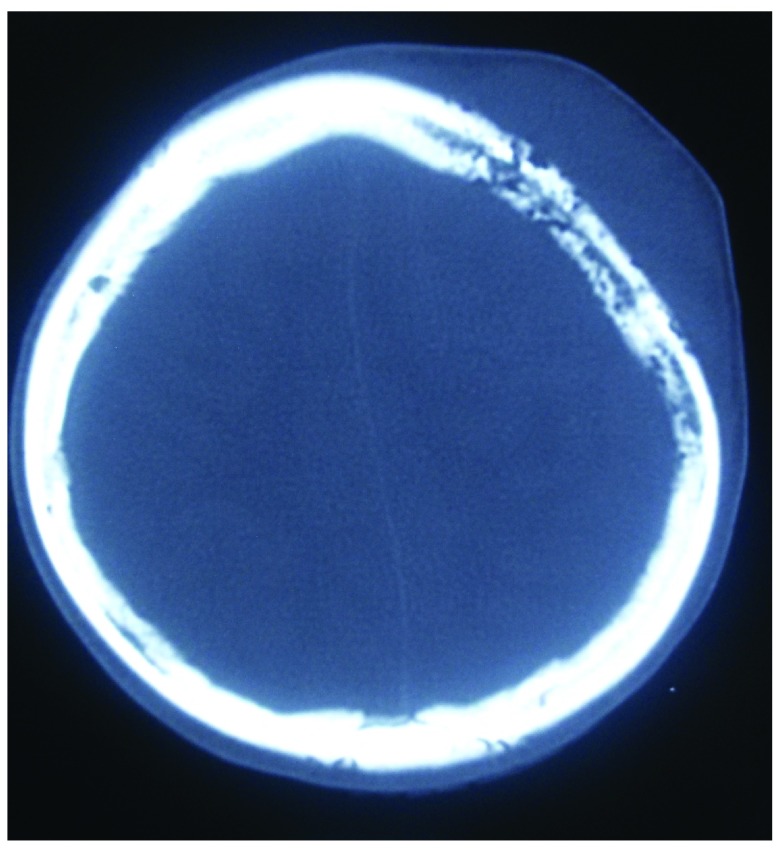
CT image showing the ‘Honeycomb’ appearance of the involved bone.

On initial examination at our ER room, the patient attained a Glasgow Coma Scale (GCS) of E2M4V2 with anisocoria on the left side. There were two scalp swellings on the left parietal and the frontal regions ( Figure 1) which were soft and fluctuant. Serology performed was negative for human immuno-deficiency virus (HIV) and hepatitis B and C. Computed tomography (CT) scan of the head was performed, revealing a dural-based hyperintense lesion on the frontal and parietal region with subfalcine herniation ( Figure 2 and Figure 3) and honeycomb appearance of the involved bone ( Figure 4). Ultrasonography of the abdomen revealed no significant lymph nodes.

**Figure 5.**
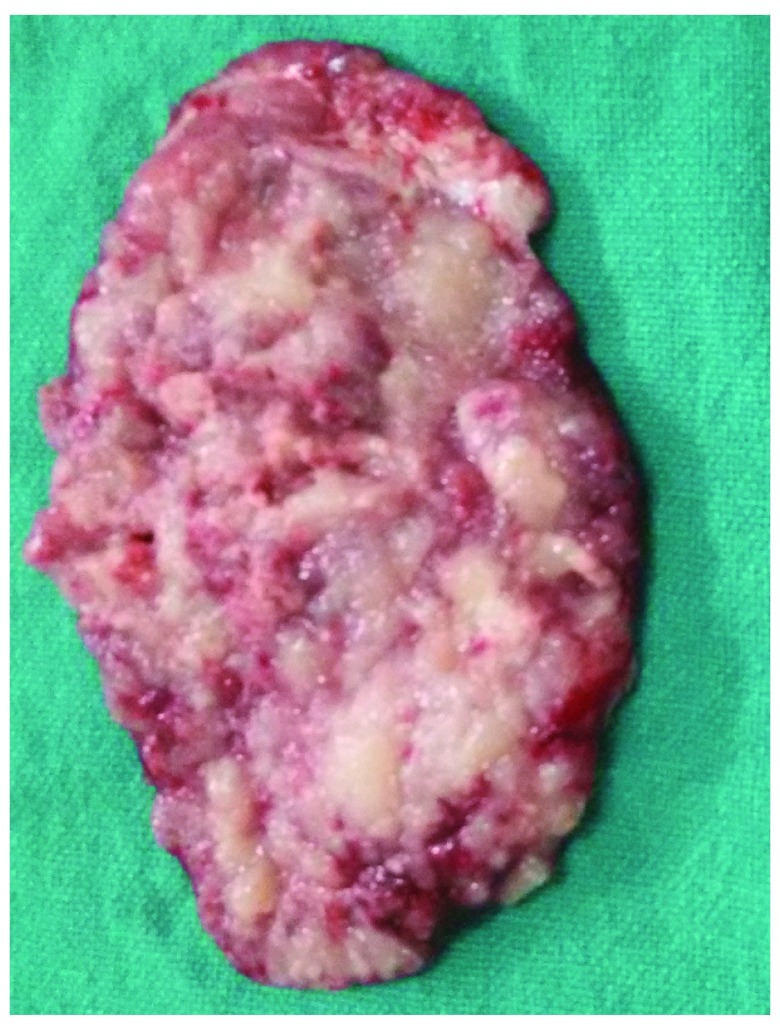
Photograph of the involved dura.

**Figure 6.**
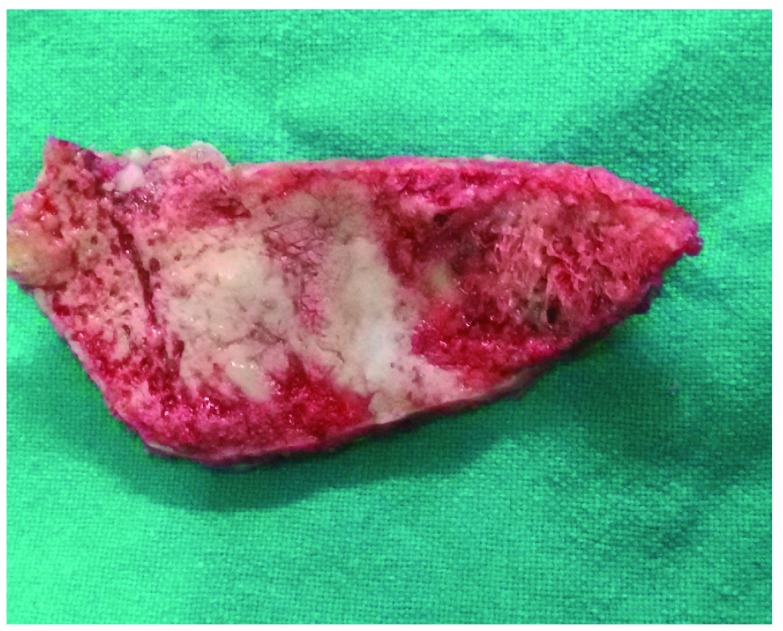
Photograph of the involved bone showing the typical ‘honeycomb’ appearance.

**Figure 7.**
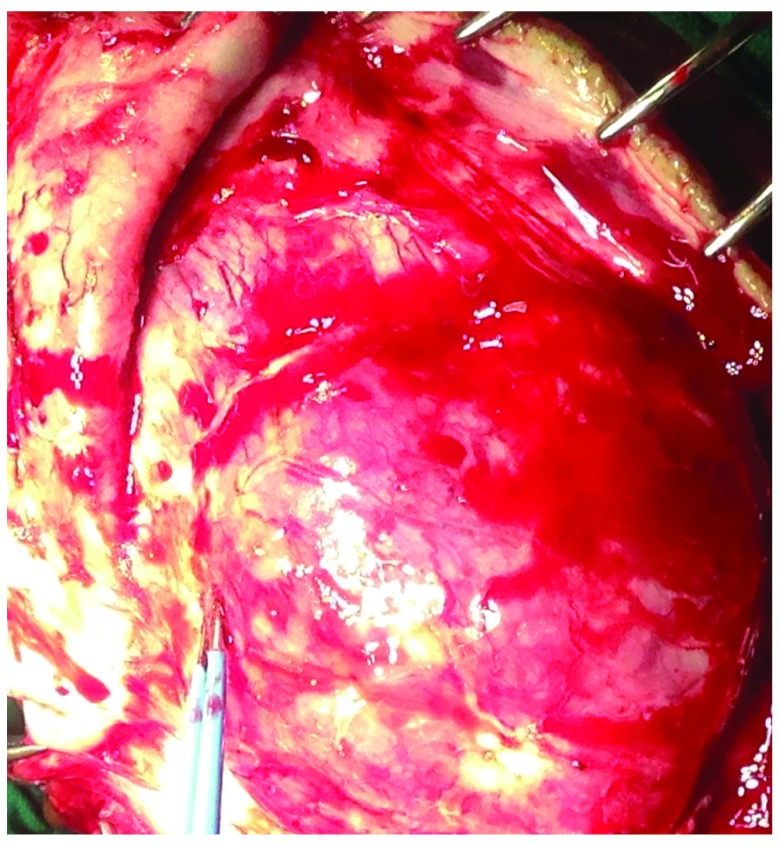
Intraoperative photograph showing the extra-calvarial extension of the lesion.

**Figure 8.**
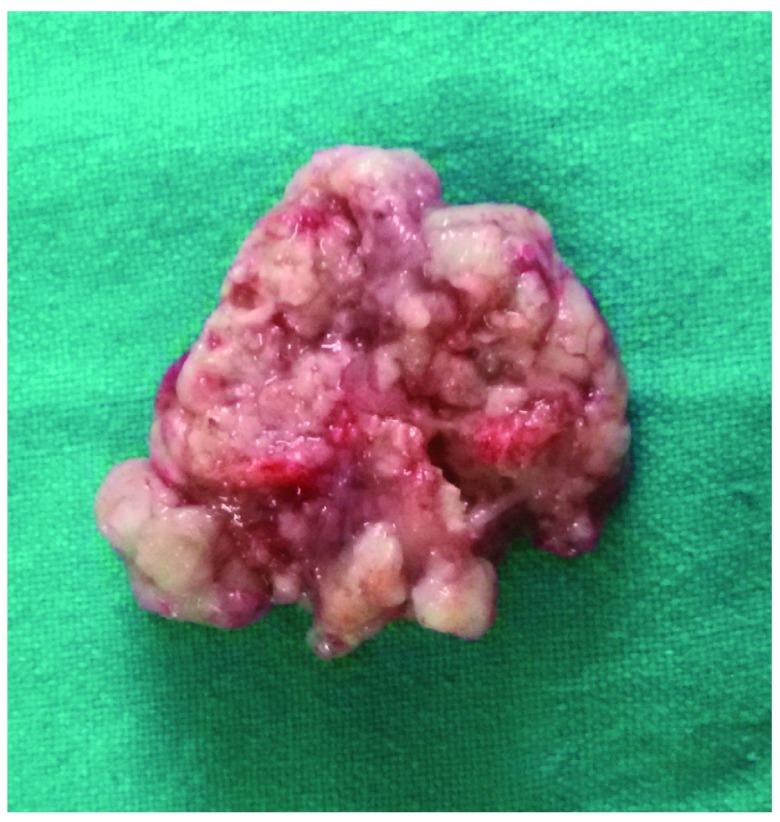
Photograph showing the portion with intradural extension.

**Figure 9.**
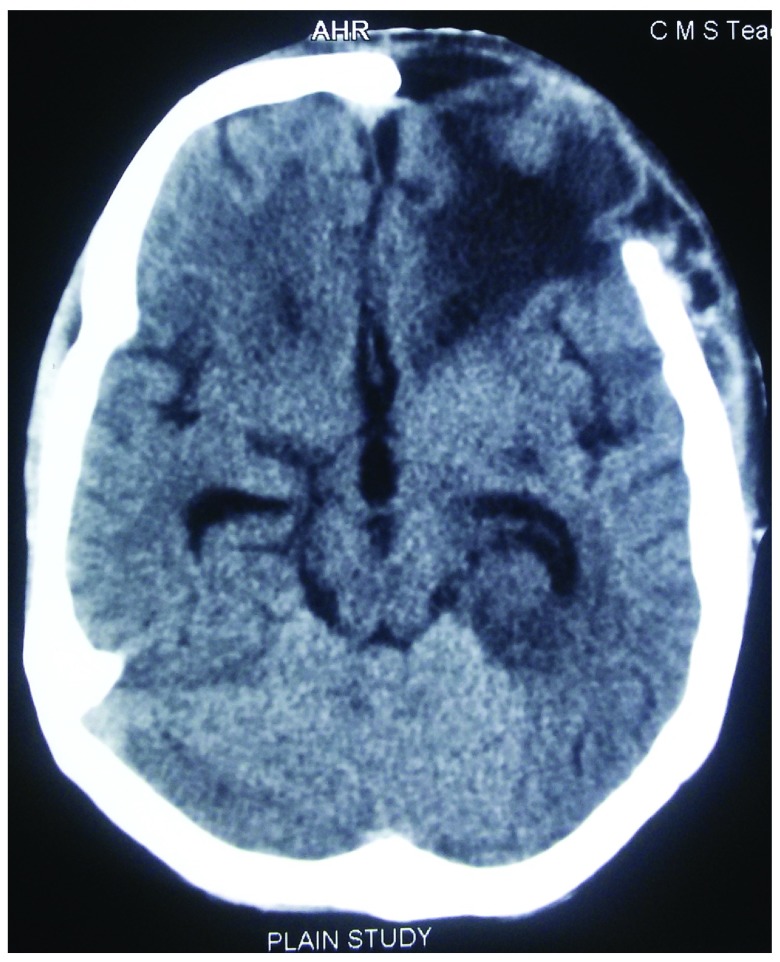
Post-operative CT image showing gross excision of lesion with resolution in mass effect.

**Figure 10.**
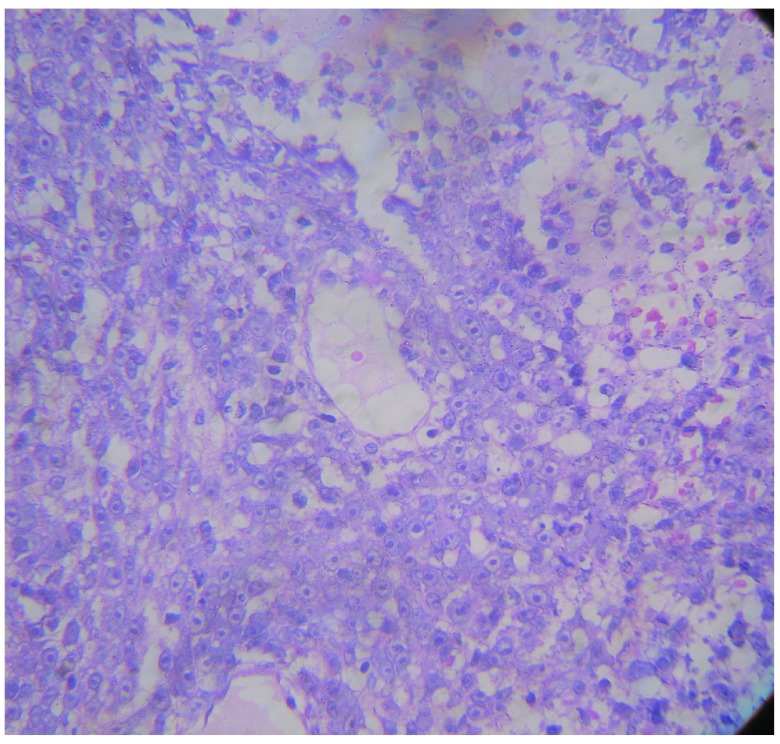
Histopathological slide showing characteristic small round blue cells with prominent nucleoli.

Because the child was already herniating, he was started on intravenous dexamethasone (4mg over 8 hours) and a single 100ml dose of 25% mannitol was given. Parents were counseled and written consent was taken for operative management. Surgery revealed a dural-based lesion ( Figure 5) that was moderately vascular, soft and friable in consistency with involved bone showing a moth-eaten appearance ( Figure 6). Both extra- and intra-dural extension ( Figure 7 and Figure 8) of the lesion was seen. Scalp lesions, involved bone, and the dural and intradural component were all excised and sent for histopathological (HPE) study. A post-operative scan showed gross excision of the lesions and absence of mass effect ( Figure 9). The HPE revealed an immunoblastic variant of diffuse large cell lymphoma ( Figure 10).

Postoperatively, the patient improved to GCS 15. The steroids were slowly tapered off as the mass effect and edema were absent on repeat CT image and also prolonged usage would hamper healing of scalp surgical wound. The patient was thoroughly counseled and then referred for free chemo- and radiation therapy in a government oncology hospital.

### Follow-up and outcomes

The patient’s GCS at 2 weeks follow-up was 4. Patient has been started on chemotherapy and is also advised for regular follow-ups.

## Discussion

Primary dural lymphoma, first described by Oberling ^5^, is an exceedingly rare disease entity. Only five cases of primary dural diffuse large B-cell lymphoma have been described so far with a median age at diagnosis of around 50 years ^1^. Trauma, inflammation and viral infection have been postulated as probable causes ^6^. The symptoms of the disease are variable and non-specific. The radiological findings are indistinguishable from other dural-based lesions such as meningiomas and hemangiopericytomas ^2^. Since the prognosis of intracranial DLBCL is favourable ^4^, it is important to make a correct and timely diagnosis. Rapid progression of the symptoms, lytic lesions on the bone and restricted diffusion in magnetic resonance imaging (MRI) may provide additional clues to the diagnosis. In cases where there are no obvious neurological symptoms, it may be advisable to take a needle biopsy of the scalp tumor as described by Ochiai *et al.* ^7^. There has been no consensus on the correct treatment protocol in the management of dural large-cell lymphoma due to a paucity of cases ^3^. Previous cases have been treated with tumor resection followed by cyclophosphamide, hydroxy-doxorubicin, oncovin (vincristine) and prednisone (CHOP) with or without rituximab- or methotrexate-based chemo regimes. Additional radiation was also tried in some cases ^1^. This case is the youngest age where the entity has been observed and showed good recovery despite initial presentation with herniation syndrome. Therefore, we suggest that maintaining a high index of suspicion and timely intervention is the key to better outcome in the patients.

## Consent

Written informed consent for publication of their clinical details and images was obtained from the father of the patient.
