# Supplementary material for: Case Report: Primary dural based diffuse large B-Cell lymphoma in a 14 year-old boy
Source: F1000Res. 2015 Mar 25;4:78. [Version 1] doi: 10.12688/f1000research.6269.1 (PMC4482212; doi:10.12688/f1000research.6269.1)
Supplement: Peer review history

# Peer review: Referee response for version 1

**DOI:** 10.5256/f1000research.6724.r9101

**Published:** 2015-06-24

**Authors:** Amit Thapa

**Affiliations:** 1 Kathmandu Medical College, Kathmandu, Nepal

The authors Munakomi *et al. *have written a nice case report which highlights a rare disease. Diffuse large cell, mixed and immunoblastic lymphomas of B cell origin should be considered together as aggressive lymphomas ^1^ which unlike MALT associated primary dural lymphomas are not indolent. However certain facts need to be discussed.

1. Even though rare, this case is not the sixth reported in literature. Other extra axial locations noted are orbit ^2^, Meckel cave ^3^^,^^4^, parasellar ^5^^,^^6^, zygoma ^2^^,^^7^, cerebellopontine angle ^8^. In 2010, 93 cases with dural based lymphomas with 10 having extra CNS (systemic involvement) had been reported ^9^.
2. Tumor immunophenotyping using flow cytometric analysis (CD5, CD10, CD19, and CD20) and by histochemical evaluation of the lymphoid cells is mandatory. These exhibit strong immunoreactivity both to leukocyte common antigen and to B-cell marker (L26). No staining should be noted for chromogranin, synaptophysin, or the epithelial markers AE1 and AE3 ^1^. 
3. Even though financial restriction is of concern in our country, contrast enhanced CT chest and abdomen should be the protocol to see for lymph node involvement rather than insensitive USG study.
4. This may be a typo error, however to correct the same: Mannitol comes in 20% and not in 25% in Nepal. 
5. Image (figure 9) show a limited craniotomy causing brain herniation from the site of craniectomy and has been taken at a level lower than lesion previously shown in Figure 2. To allow comparison, it is suggested to provide CT images  of same level or plane.
6. Differential diagnosis should include meningiomas, metastases,extradural abscesses with osteomyelitis or extradural hematomas. Clues for diagnosis include a rapid progression of symptoms with permeative growth pattern with large soft tissue component in galeal and extradural compartment, restricted diffusion on MRI, and the presence of bone lytic lesions on CT, limited periosteal reaction and an angiographically avascular lesion except for blush or vascular encasement ^1^^,^^10^.
7. The treatment of choice for primary CNS lymphoma is chemotherapy (Adriamycin containing CHOP protocol) followed by radiation of the involved field and surgical excision is limited to conditions where mass effect is life threatening.

Recognising this entity is important because an early diagnosis and rapid initiation of treatment, in certain cases with sole chemotherapy, is associated with a high response rates and favourable outcome ^10^.

I have read this submission. I believe that I have an appropriate level of expertise to confirm that it is of an acceptable scientific standard.

## References

[1] GalarzaMGazzeriRElfekyHAJohnsonRR: Primary diffuse large B-cell lymphoma of the dura mater and cranial vault. Case report and literature review. *Neurosurg Focus*.2006;21(5) :e10 1713411210.3171/foc.2006.21.5.11

[2] PaigeMLBernsteinJR: Transcalvarial primary lymphoma of bone. A report of two cases. *Neuroradiology*.1995;37(6) :456-458 747785710.1007/BF00600092

[3] Abdel AzizKMvan LoverenHR: Primary lymphoma of Meckel’s cave mimicking trigeminal schwannoma: case report. *Neurosurgery*.1999;44(4) :859-862 1020131210.1097/00006123-199904000-00096

[4] ArticoMSalvatiMRacoAInnocenziGDelfiniR: Primary Meckel’s cave lymphoma. A case and review of the literature. *Neurochirurgie*.1992;38(6) :368-371 1306893

[5] OyamaHNaganeMShibuiSNomuraKMukaiK: Skull base malignant lymphoma: a case report and review of the literature. *Jpn J Clin Oncol*.1992;22(2) :131-135 1619780

[6] Roman-GoldsteinSMJonesADelashawJBMcMenomeySNeuweltEA: Atypical central nervous system lymphoma at the cranial base: report of four cases. *Neurosurgery*.1998;43(3) :613-615 973331710.1097/00006123-199809000-00119

[7] NgYYCookJAMcRaeRD: Lymphoma of the zygoma: an unusual cause of unilateral facial swelling. *J Otolaryngol*.1990;19(5) :315-318 2262949

[8] BercianoJJimenezCFigolsJFerreresJCCombarrosOArjonaRPascualJ: Primary leptomeningeal lymphoma presenting as cerebellopontine angle lesion. *Neuroradiology*.1994;36(5) :369-371 793617710.1007/BF00612120

[9] MatmatiKMatmatiNHannunYARumboldtZPatelSLazarchickJStuartRGiglioP: Dural MALT lymphoma with disseminated disease. *Hematology Reports*.2010;2(1) : 10.4081/hr.2010.e10e10 10.4081/hr.2010.e10 22184513PMC3222263

[10] BritoABCReisFde SouzaCAVassalloJLimaCSP: Intracranial primary dural diffuse large B-cell lymphoma successfully treated with chemotherapy. *Int J Clin Exp Med*.2014;7(2) :456-460 24600506PMC3931605

# Peer review: Referee response for version 1

**DOI:** 10.5256/f1000research.6724.r8460

**Published:** 2015-05-15

**Authors:** Guoyi Gao

**Affiliations:** 1 Department of Neurosurgery, Renji Hospital, Shanghai Institute of Head Trauma, Shanghai, China

Primary dural lymphoma is the disease to most neurosurgeons and this case report provided details of a young patients' clinical treatment including diagnosis, surgery, and following chemotherapy and radiotherapy choices. It is worth indexing to improve the management of dural lymphoma.

I have read this submission. I believe that I have an appropriate level of expertise to confirm that it is of an acceptable scientific standard.
